# The Gastrointestinal Stromal Tumor (GIST) of a Pancreatic Cyst

**DOI:** 10.7759/cureus.26197

**Published:** 2022-06-22

**Authors:** Rasiq Zackria, Vijay Jayaraman

**Affiliations:** 1 Gastroenterology and Hepatology, Sunrise Health Graduate Medical Education (GME) Consortium, Las Vegas, USA; 2 Gastroenterology and Hepatology, Comprehensive Digestive Institute of Nevada, Las Vegas, USA

**Keywords:** pancreatic extragastrointestinal stromal tumor (egist), gastrointestinal stromal tumor (gist), pancreatic pathology, fine-needle aspiration, endoscopic ultrasound (eus)

## Abstract

Gastrointestinal stromal tumors (GISTs) are rare tumors and rarely occur outside of the gastrointestinal (GI) tract. When GISTs originate from outside the GI tract, they are called extra-gastrointestinal stromal tumors (EGISTs). In this article, we discuss a case of a 74-year-old woman who presented due to a growing pancreatic lesion on imaging and was subsequently diagnosed with pancreatic EGIST on endoscopic ultrasound with fine-needle aspiration.

## Introduction

Gastrointestinal stromal tumors (GISTs) are rare tumors accounting for less than 1% of all gastrointestinal (GI) tumors [1,2]. Although rare, they are the most common mesenchymal tumors in the GI tract. These tumors tend to arise more frequently in the stomach and small intestine. In fewer than 5% of cases, they primarily originate from outside the GI tract. In the cases where GISTs originate from outside the GI tract, they are called EGISTs (extra-gastrointestinal stromal tumors). EGISTs are extremely rare stromal tumors that have no connection to the wall or show no involvement with the peritoneal surface of the GI tract [3]. While EGISTs usually arise from the omentum, mesentery, and retroperitoneum [4] adjacent to the stomach and intestine, primary pancreatic EGISTs are extremely rare and account for less than 5% of EGISTs [5]. A little over 30 cases of pancreatic EGISTs have been reported in the literature [3].

## Case presentation

**Figure 1:**
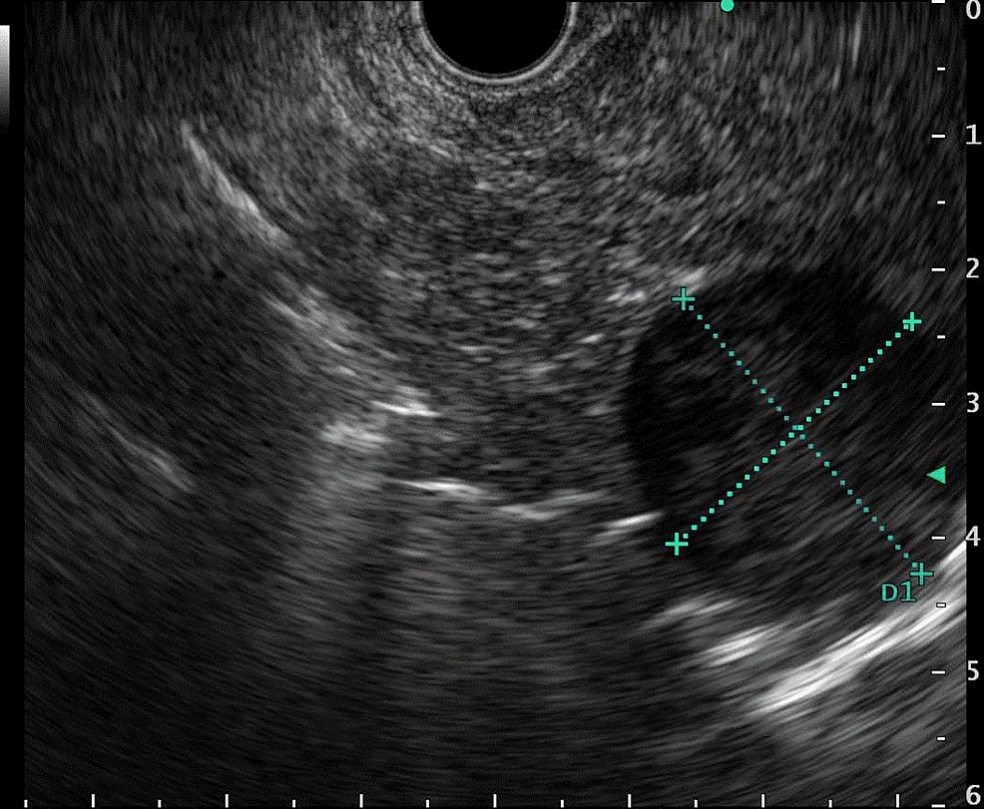
Endoscopic ultrasound demonstrating a well-circumscribed 2.9 cm x 2.5 cm hypoechogenic mass arising from the uncinate process of the pancreas

A 74-year-old asymptomatic woman with a medical history significant for hypertension, type 2 diabetes mellitus, tobacco use disorder, and kidney cyst presented to our gastroenterology clinic due to abnormal findings on imaging of her abdomen. About a year prior to presentation, the patient underwent magnetic resonance imaging (MRI) of her abdomen for follow-up of her kidney cyst which demonstrated a 2.2-centimeter (cm) renal cyst and a 2-millimeter (mm) pancreatic cyst. Follow-up MRI showed a 2.3 cm x 2.5 cm well-circumscribed pancreatic head mass. There was no pancreatic duct dilation, biliary obstruction, or peripancreatic lymphadenopathy. Due to the relatively rapid lesion growth, the patient underwent endoscopic ultrasound (EUS) with fine-needle aspiration (FNA) for further evaluation. EUS showed a well-circumscribed 2.9 cm x 2.5 cm hypoechogenic mass (Figure 1) that appeared to arise from the uncinate process of the pancreas but was restricted to the pancreatic parenchyma.

**Figure 2:**
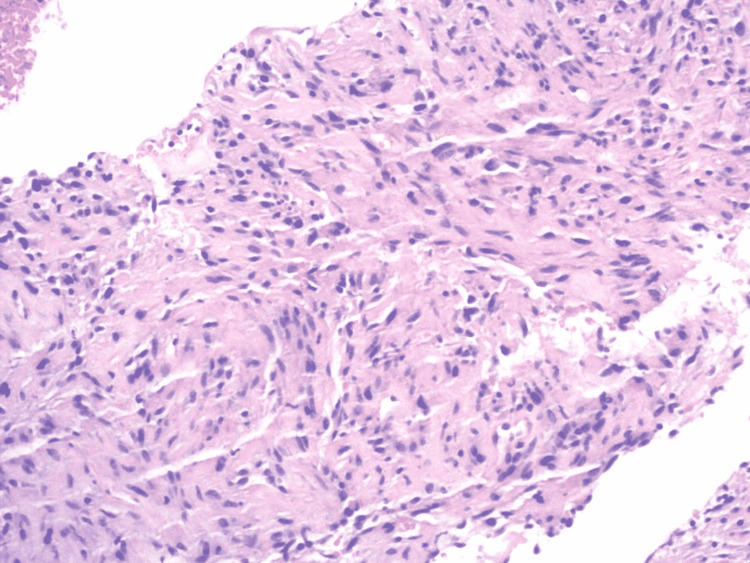
H&E stain showing disorganized and bland spindle cells associated with abundant eosinophilic cytoplasm H&E: Hematoxylin and eosin.

The rest of the pancreatic parenchyma and pancreatic duct was unremarkable without evidence of cysts, masses, pancreatic lymphadenopathy, or chronic pancreatitis. A 22-gauge core biopsy needle was introduced through the endoscope, and four passes were made for the FN biopsy of the lesion. The lesion did not have a classic appearance for adenocarcinoma and did not appear to invade any surrounding structures. An immediate assessment of the obtained specimen suggested benign spindle-shaped cells. The spindle cells appeared to be disorganized, bland, and associated with abundant eosinophilic cytoplasm on further pathologic evaluation (Figure 2).

**Figure 3:**
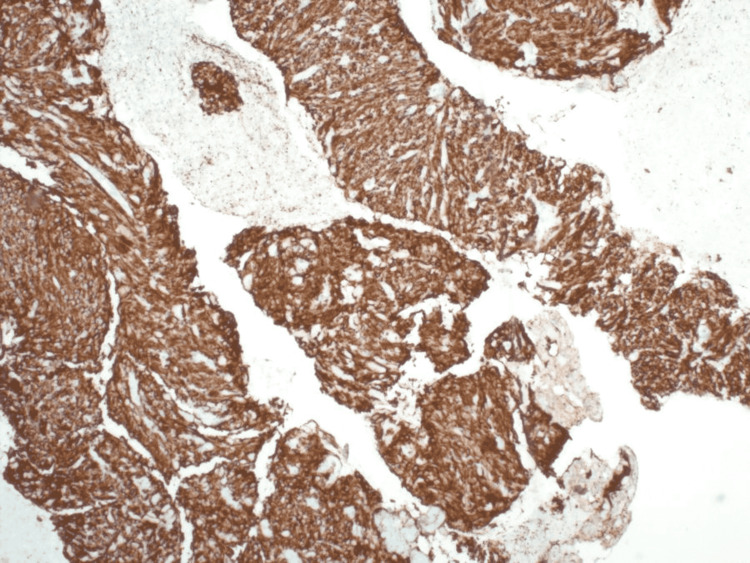
Immunohistochemistry stain showing the atypical cells diffusely positive for CD117, confirming the diagnosis of a GIST CD117: Cluster of differentiation 117 (Proto-oncogene c-KIT); GIST: Gastrointestinal stromal tumor.

No nuclear pleomorphism, extensive mitotic activity, or necrosis was seen. Immunohistochemistry showed that the atypical cells were strongly and diffusely positive for CD117 (Figure 3) and CD34; there was weak staining with smooth muscle actin (SMA).

The stains for S-100, desmin, and pankeratin (AE1/AE3) were negative. The morphologic and immunohistochemistry findings were most consistent with the GIST of the uncinate process of the pancreas. The patient was referred to oncology and surgery for further management. Further molecular analysis demonstrated significant benefit with tyrosine kinase inhibitor therapy. The patient was started on imatinib therapy for the treatment of her EGIST with possible surgical intervention in the near future.

## Discussion

Pancreatic EGISTs are usually found incidentally in imaging studies with definite diagnoses based on the immunohistochemical examination. Mostly, these tumors are asymptomatic, with symptoms present depending on the tumor location and dimension in the pancreatic tissue [6,7]. The most reported symptoms are abdominal discomfort and weight loss. In contrast, other findings including fatigue, fever of unknown origin, anemia, portal vein thrombosis, and jaundice have also been reported in some cases [5]. Diagnostic studies for pancreatic masses include biomarkers such as carbohydrate antigen 19-9 and carcinoembryonic antigen (CEA) [8]. In addition to these biomarkers, radiological, histopathological, immunohistochemical, and genetic testing are useful diagnostic studies in the diagnosis of a pancreatic mass [8]. However, the diagnostic value of the tumor markers is limited for diagnosing pancreatic EGISTs and is rarely used [2]. Abdominal computed tomography (CT), MRI, and EUS are the most frequently employed radiologic techniques to determine tumor location, dimensions, margin irregularity, metastasis, or invasion of surrounding structures, and resectability [9]. Previously, ultrasound or CT-guided biopsy was used for diagnosis; however, the increasing emergence of EUS as a valuable diagnostic and therapeutic tool has allowed for simultaneous evaluation and tissue acquisition of solid or cystic pancreatic masses [5].

Histopathologically, GISTs can be classified into the spindle (70%), epithelioid (20%), or mixed (10%) type; most pancreatic EGISTs consist of spindle cells [2]. Given this finding, the differential diagnosis of leiomyoma, leiomyosarcoma, liposarcoma, rhabdomyosarcoma, schwannoma, fibromatosis, inflammatory fibroid polyps, solitary fibrous tumor, and malignant fibrous histiocytoma should be considered [10]. Among the typical immunohistological staining features of EGISTs, CD117 - a proto-oncogene c-KIT known for encoding the receptor tyrosine kinase protein - is the most well known. c-KIT is a recently discovered member of the KIT transmembrane receptor family (for binding tyrosine kinase enzymes), whose receptor is an epitope that can be stained. Approximately, 95% of tumors defined as GIST or EGIST stain CD117-positive. Additionally, GISTs stain positive for CD34 (60%-70%), heavy caldesmon (80%), SMA (30%-40%), S100 (5%), and desmin (<5%) [2,5,7,11-13].

While tyrosine kinase inhibitors, such as imatinib, can be used for neoadjuvant and adjuvant therapy with improved survival, surgical resection is the definitive treatment for pancreatic EGIST [1]. The goal of surgical resection is to achieve clear margins with complete resection. The location of the pancreatic EGIST determines the optimal type of surgical resection. While pancreaticoduodenectomy is the optimal treatment option for pancreatic head tumors, distal pancreatectomy is preferred in pancreatic tail tumors [14]. To reduce the tumor size of large-size GISTs, imatinib can be used as neoadjuvant therapy for large-size GISTs [15]. It has also been shown to reduce tumor size, increase the rate of complete resection of the tumor, and help to improve prognosis [15].

## Conclusions

Our case highlights the importance of pursuing EUS with FNA evaluation of any suspected pancreatic lesion. Although pancreatic EGISTs are uncommon tumors, they must be considered in the differential diagnosis of pancreatic lesions. Unfortunately, the lack of comprehensive case reports on pancreatic EGISTs and long-term follow-up studies limit the available information on EGISTs.
